# Feasibility of near-infrared spectroscopy for species identification and parasitological diagnosis of freshwater snails of the genus *Biomphalaria* (Planorbidae)

**DOI:** 10.1371/journal.pone.0259832

**Published:** 2021-11-11

**Authors:** Vanessa Valladares, Célio Pasquini, Silvana Carvalho Thiengo, Clélia Christina Mello-Silva

**Affiliations:** 1 Evaluation and Promotion of Environmental Health Laboratory, Instituto Oswaldo Cruz-Fiocruz, Rio de Janeiro, RJ, Brazil; 2 National Institute of Advanced Analytical Sciences and Technologies (INCTAA), State University of Campinas—UNICAMP / Chemistry Institute, Campinas, SP, Brazil; 3 Laboratory of Malacology, Instituto Oswaldo Cruz- Fiocruz, Rio de Janeiro, RJ, Brazil; George Washington University, UNITED STATES

## Abstract

Near Infrared Spectroscopy (NIRS) has been applied in epidemiological surveillance studies of insect vectors of parasitic diseases, such as the Dengue’s mosquitoes. However, regarding mollusks, vectors of important worldwide helminth diseases such as schistosomiasis, fascioliasis and angiostrongyliasis, NIRS studies are rare. This work proposes to establish and standardize the procedure of data collection and analysis using NIRS applied to medical malacology, i.e., to mollusk vectors identifications. *Biomphalaria* shells and live snails were analyzed regarding several operational aspects, such as: moisture, shell side and position of the live animal for acquisition of NIR spectra. Representative spectra of *Biomphalaria* shells and live snails were collected using an average of 50 scans per sample and resolution of 16 cm^-1^. For shells, the sample should first be dried for a minimum of 15 days at an average temperature of 26±1°C, and then placed directly in the equipment measurement window with its left side facing the light beam. Live animals should be dried with absorbent paper; placed into a glass jar, and analyzed similarly to the shells. Once standardized, the technique was applied aiming at two objectives: identification of *Biomphalaria* using only the shells and parasitological diagnosis for *Schistosoma mansoni* infection. The discrimination of the three *Biomphalaria* species intermediate hosts of *S*. *mansoni* only by shell has technical limit due to the scarcity of organic material. Nevertheless, it was possible to differentiate *B*. *straminea* from *B*. *tenagophila* and *B*. *glabrata* with 96% accuracy. As for the parasitological diagnosis, it was possible to differentiate infected mollusks shedding *S*. *mansoni* cercariae from the non-infected ones with 82, 5% accuracy. In conclusion, the Near Infrared Spectroscopy (NIR’s) technique has proven to be an innovative and sound tool to detect infection by *S*. *mansoni* in the different species of *Biomphalaria* intermediate hosts.

## Introduction

Near-infrared Spectroscopy (NIRS) is a technique of spectroscopy that assesses the vibrational energy levels associated to chemical bonds present in different molecules within a wavelength range 750–2500 nm. This technique is of simple implementation and delivers methods to acquire spectral data related to qualitative and quantitative characteristics of the biomass of living organisms through the interaction of the electromagnetic waves with the sample chemical species. With a potential acquisition speed of 50 scans per minute, this technique does not require any intensiveness prior to treatment of the sample, it is non-invasive and non-destructive, and does not generate dangerous residues, which means that it is widely considered to be an environmentally-friendly analytical technique [1, 2].

Near Infrared Spectroscopy has been applied as an exploratory analytical tool since 1938 [2, 3], but only recently its potential use in biological sciences has been investigated, with a variety of applications in biodiversity studies such as ecology, taxonomy, biochemistry and parasitology [4–8]. In the specific case of mollusks, IRS has been applied in the mid range for the investigation of phenomena such as the process of shell formation (biomineralization) and its diagnostic markers [9–16], the composition of the organic matrix of the shell [17], and the identification of chitin in shells from archaeological sites [18]. Regarding near infrared, only one paper has been published so far approaching its use in mollusks, aiming at identifying *Biomphalaria* species, intermediate hosts of *Schistosoma mansoni*, collected from the field as well as the degree of contamination of the freshwater environments they inhabit [19].

Given the potential applications of NIRS for identification of species that act as intermediate host of *S*. *mansoni* using live snails, we decided to investigate the possibility of discriminating *Biomphalaria* species using only the shells as well as to differentiate *S*. *mansoni* infected mollusks from non-infected ones. Therefore, this work proposes to present a standardized protocol for data collection and analysis in medical malacology using NIR for distinguishing different *Biomphalaria* species and detecting *S*. *mansoni* infection in those snails.

## Materials and methods

Considering that rare studies have used FT-NIR to analyze *Biomphalaria* specimens, it was necessary to evaluate the procedures to standardize the spectrophotometric analysis of the three *Biomphalaria* species. Therefore, we followed Vance *et al*. [4] and previous methods adopted to study other invertebrates such as mosquitoes [5, 6] and bivalve mollusks [20] to apply NIR spectroscopy for *Biomphalaria* species. Several aspects related to the measurement protocol were evaluated, such as: the best side of the shell for data acquisition, the influence of shell’s moisture on the sample spectrum, and the feasibility of collecting representative spectra from live animals infected and non-infected by *S*. *mansoni*.

### Snail strain (shells and live animals)

To standardize the FT-NIR technique for the analysis of *Biomphalaria*, 120 shells (40 shells per species) and 250 alive animals (50 infected and 200 non-infected snails) were analyzed. All the snails (4–5 weeks age) were obtained from the National Reference Laboratory in Schistosomiasis and Malacology (LRNEM) at Oswaldo Cruz Institute/Oswaldo Cruz Foundation -IOC/FIOCRUZ in Rio de Janeiro. Specimens were measured until their shell’s diameter became in the range of 8–10 mm, i.e. adult snails, in order to avoid biochemical changes related to the ontogenetic growth of the animals. After the onset of sexual maturity, i.e., the first oviposition, the biochemical composition of the individual becomes more stable and resembles that of the adult [21]. The snails were raised under standard conditions, where water temperature is maintained at 25 ± 3°C and room temperature at 28 ± 3°C, and fed with washed lettuce (*Lactuca sativa* L.), provided *ad libitum*.

### Near infrared spectrophotometry

The present study used an ABB Bomem MB 3600 near infrared spectrophotometer with Fourier Transformation (FT-NIR) and powder sampler, which is located in the Environmental Health Research Laboratory at IOC/FIOCRUZ. This apparatus is validated weekly to guarantee its well operating conditions, using a standard reference sample (a glass vial containing spectralon powder), through the manufacturer’s protocol. The same spectralon material was used to obtain the reference background for all spectrum acquisition. The background signal was obtained prior to the analysis of each batch of samples and after the analysis of three consecutive samples. In all analyses, 50 spectra were obtained from each specimen (shell or alive snail) from which a mean representative spectrum was calculated at a resolution of 16 cm^-1^.

### Chemometric analyses

The chemometric analyses of the spectra were run in the Unscrambler® 10.5 software where the raw spectra were pre-treated using the Savitzky-Golay smoothing procedure (31 point window and 2nd order polynomial) and the 1^st^ Savitzky-Golay derivative (15 point window and 2nd order polynomial) to minimize possible variation derived from noise and unwanted variability in the signal, which can influence the final result. The data were initially explored using a Principal Components Analysis (PCA) to verify the potential formation of sample groups, followed by a Linear Discriminant Analysis (LDA) based on the PCA scores of the spectra set to reduce the dimensionality of the data and extract the most discriminating characteristics allocating the samples into a single class [22]. The best LDA model employed 6 principal components, and a linear discriminant function.

The parameters evaluated in the present study were the position of the shell or live animal on the equipment measurement window, the minimum shell drying time, the procedure used for collection of spectra from the live animals, and the minimum number and diversity of the samples necessary for reliable chemometric analyses.

### (i) The preferred side of the shell for the best data acquisition

For this standardization, 30 shells from each species of Brazilian populations of *B*. *glabrata* from Jacobina/ state of Bahia (BA), *B*. *tenagophila* from Itamaraju (BA), and *B*. *straminea* from Jaboticatubas/state of Minas Gerais (MG) were used. Spectra were obtained from the right and left side of the shell and subsequently compared to determine the best side of the shell most favorable for species discrimination.

### (ii) The influence of shell’s moisture on the FT-NIR sample spectrum

To evaluate the influence of the moisture of the shell on NIR spectra, 10 shells of each of the three species from the same populations described in the previous section were used. The empty shells whose animals were previously removed [23], were placed on paper towels for drying in an air-conditioned room at a mean temperature of 25±2°C. The NIR spectra of the shells were obtained after eight different drying intervals (1, 3, 5, 15, 30, 60, 90, and 120 days).

### (iii) Feasibility of spectra acquisition from live infected and non infected snails by *Schistosoma mansoni*

To evaluate the possibility of collecting spectra from alive animals, 150 snails (50 of each species) were kept in separate Petri dishes prior to FT-NIR measurement. The animals were first dried superficially with absorbent paper, to remove excess water from both sides of the shell, and each snail was then placed inside a transparent glass vial with the left side facing downward.

Preliminary analysis of a parasitological diagnosis was performed on 100 specimens of *B*. *glabrata* from Belo Horizonte (MG). Half of the animals were infected individually, in a 24-well plate, with 7–10 miracidia of *S*. *mansoni*, a strain sympatric to the mollusk species. The remaining non-infected animals were analyzed as a control group.

After elimination of cercariae and verification of infection efficacy, the mollusks were numbered and their FT-NIR spectra were obtained randomly, employing the same procedure described above for alive animals.

## Results and discussion

The standardization of procedures proposed in this work proved to be an important step towards the application of NIR spectroscopy in the field of mollusk studies by providing reproducible information to differentiate *Biomphalaria* species using only shell, in addition to performing parasitological diagnosis of *S*. *mansoni* in the mollusk intermediate host.

**Fig 1:**
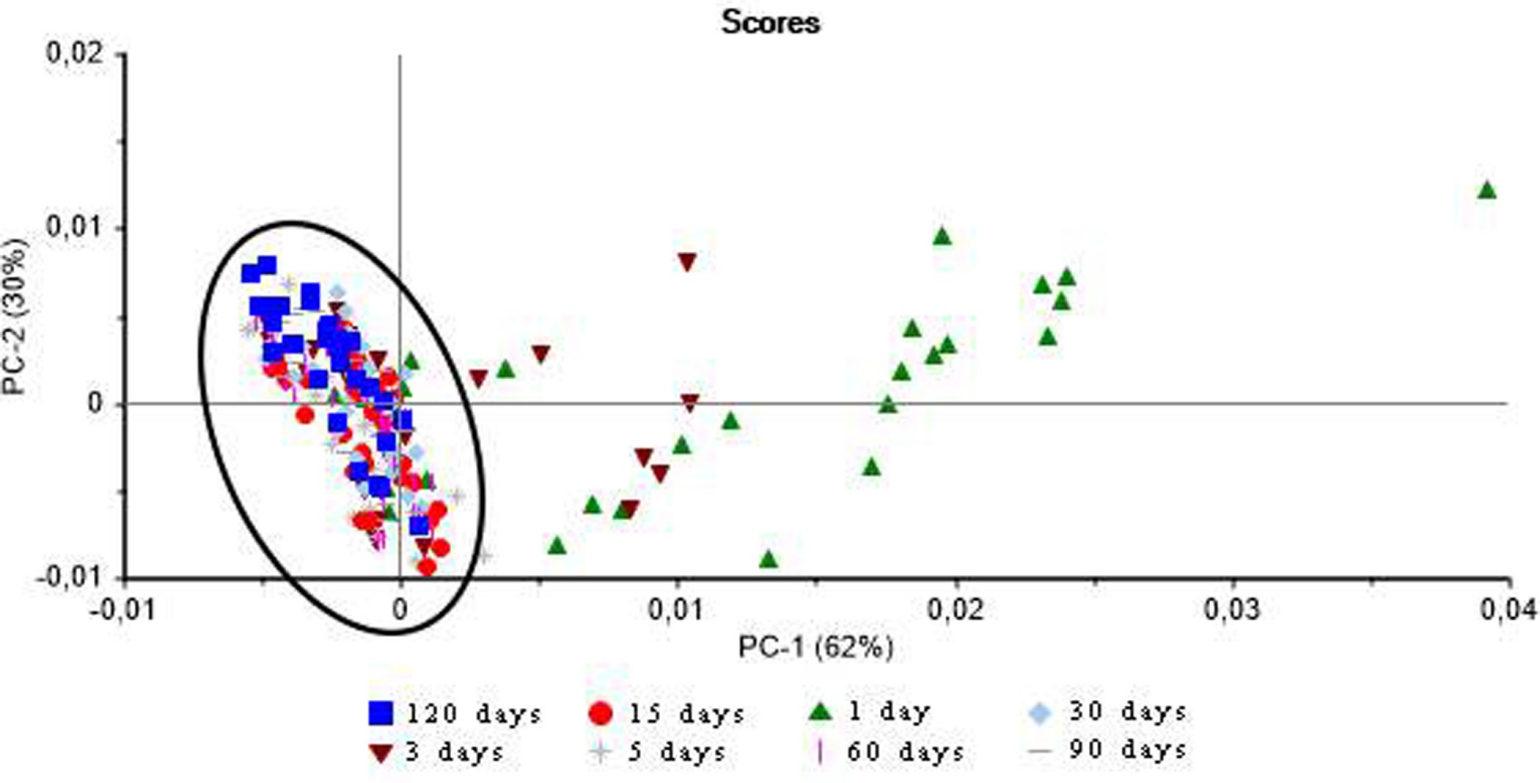
NIR spectra of the shells after different drying periods. Plot of the principal component scores showing variation in the spectra obtained from the shells of the three *Biomphalaria* species after different periods of drying. The data points circled by the ellipse represent the groups with similar results.

The influence of humidity on the results of the FT-NIR analysis was tested using a total of 30 shells, which were analyzed to observe the drying time. The spectra of the shells varied according to the duration of the drying period. The PCA results of the data on the three *Biomphalaria* species showed that the samples dried for 1 or 3 days were much more widely dispersed that those dried for 5 days or more (Fig 1). In addition, a higher number of correct hits for the classification of the species (more than 90%) were obtained using the LDA classification method and the data collected after 15 or more draying days.

**Fig 2:**
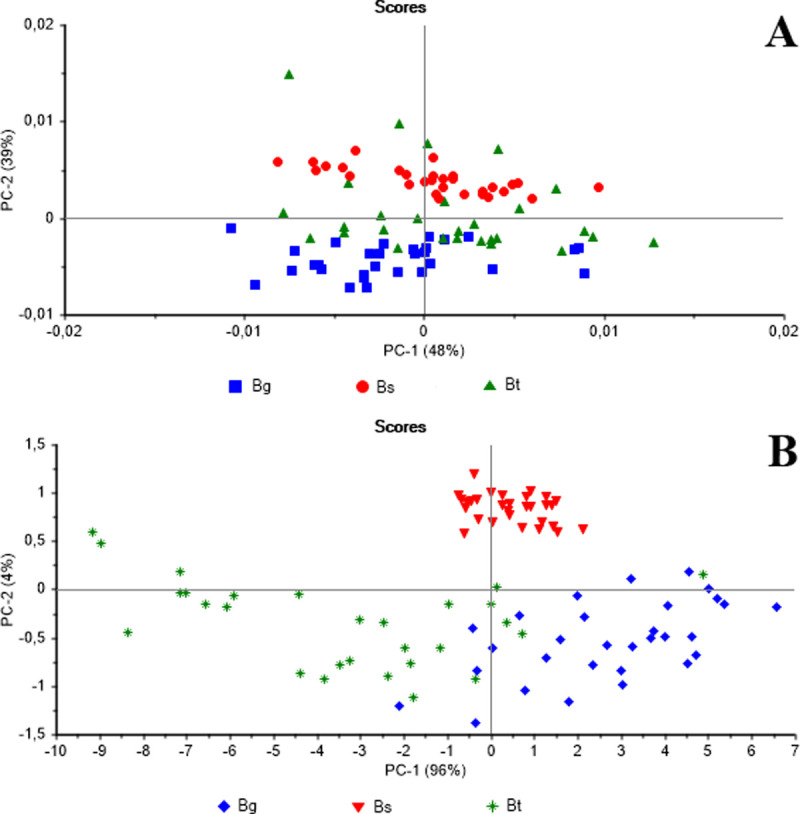
Plots of the principal components scores of the NIR spectra of the *Biomphalaria* shells dried for 15 days. (A) NIR spectra obtained from the right side of the shell. (B) NIR spectra obtained from the left side of the shell. Bg = *Biomphalaria glabrata*; Bt = *Biomphalaria tenagophila*; Bs = *Biomphalaria straminea*.

Each shell was positioned in the probing window of the FT-NIR spectrophotometer for measurement on both its right and left sides. The scores for the first two principal components are shown in Fig 2. For the right side of the shells, principal component 1 (PC-1) accounts for less than half (48%) of the variance of the pretreated spectra data set. Considering the first two components (87% of the data variance captured) the separation among the three species is not observed. On the other hand, for the left side, PC-1 captured approximately 96% of the spectral data. Furthermore, the scores for the first two components (100% captured variance) reveal that the three species are well grouped with PC-1 distinguishing *B*. *glabrata* from *B*. *tenagophila*, and PC-2 distinguishing these two species from *B*. *straminea*. Therefore, the spectra obtained from the left side of shells shows a high potential to distinguish the three species of mollusks.

It is important to note here that, in all the three species, the shell opens to the left side, which means that the animal’s internal organs also face this side [24]. Given this, all the subsequent procedures, involving both alive animals and shells, were focused on the left side of the shell.

With the standardization of the left side of the shell as the best side and the drying period of 15 days, the validation of the LDA method applied to the samples shown in Fig 2B achieved 96% accuracy in the classification of the three species. Shells can be used as one of the morphological characteristics to differentiate species. However, dissection and characterization of structures of the reproductive and excretory apparatus are also required for correct taxonomic identification [24]. These preliminary results demonstrate the feasibility of differentiating *Biomphalaria* species using NIR.

The possibility of different composition of shells, the direct participation of shells in the mollusk metabolism and the preservation of shells over time make the results observed even more interesting. Studies regarding *Biomphalaria* shells are restricted to the formation and composition of *B*. *glabrata* shells [17, 25–27]. As for the structure and composition, the organic matrix present in the periostracum is what is probably observed in the NIR spectra due mainly to the presence of lipids and proteins such as dermatopontine [26]. In addition, the shells of *Biomphalaria* spp. participate directly in the regulation of calcium metabolism [28], which can also contribute to species differentiation.

The similarity of the scores between *B*. *glabrata* and *B*. *tenagophila* indicate that the chemical phenotypes of the two species are the most similar when compared to *B*. *straminea*. Although Vidigal et al. [29] indicated that there is considerable genetic distance between these first two species through phylogenetic analysis of the ITS2 molecular marker, this fact may be related to the morphological and biological similarities of *B*. *glabrata* and *B*. *tenagophila* and the possible potential for hybridization [23, 30]. Considering that the shells composition reflects the metabolic projections of the animal, it was expected that the three species could be differentiated using the shells alone, possibly associated with the chemical and protein arrangement of the organic matrix of the shells of these animals [26, 28, 31].

This study shows the feasibility of differentiating *B*. *straminea* from *B*. *tenagophila* and *B*. *glabrata*. It is known that the three species have biological and physiological characteristics distinct from each other, help them to adapt to different environments. Consequently, mollusk species identification is relevant to epidemiological studies related to the transmission of *Schistosoma* spp [23, 32–35].

**Fig 3:**
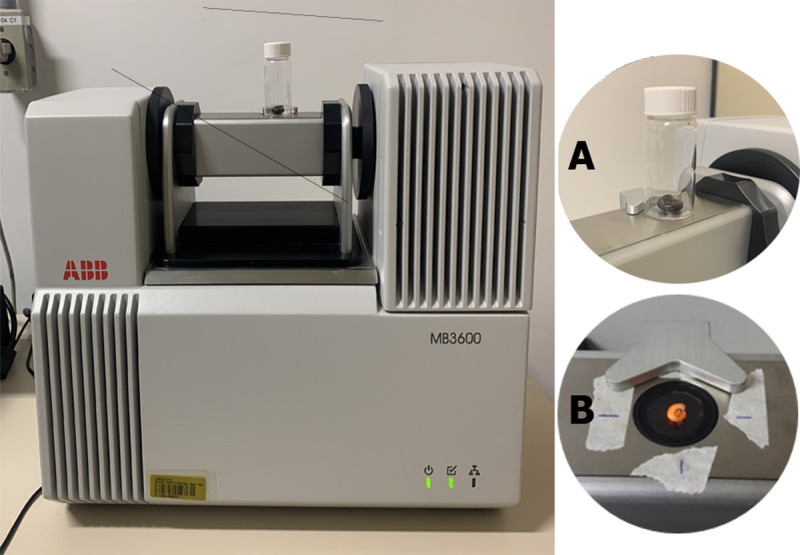
Near Infrared Spectrophotometer equipment. (A) Glass vial containing a live specimen of *Biomphalaria glabrata* ready to be measured by the FT-NIR through the window of the apparatus. (B) Dry shell of *Biomphalaria glabrata* placed directly into the FT-NIR apparatus.

As for the use of NIR spectroscopy on alive mollusks, the measurement protocol was standardized by collecting the spectra considering the previous results for shells and the symmetry of the internal organs, placing the animal with the left side down. The vial with the snail was placed directly into the window of the apparatus to obtain the spectra (Fig 3A). This step is slightly different from that of the analysis of the dry shells, in which the specimen is placed directly into the FT-NIR apparatus (Fig 3B). The spectra of the alive animals were collected with the animals being placed randomly in the window, which favors the reliability of the data collection and analysis.

After the collection of the spectra, the animals were kept separately in 10 mL beakers containing dechlorinated water. Beakers were grouped by species. The animals remained alive and were able to move, feed, and reproduce normally for at least 12 months after the analysis, as observed in normal unexposed snails. This indicates that exposure of snails to the tested Infrared did not adversely affect them.

**Fig 4:**
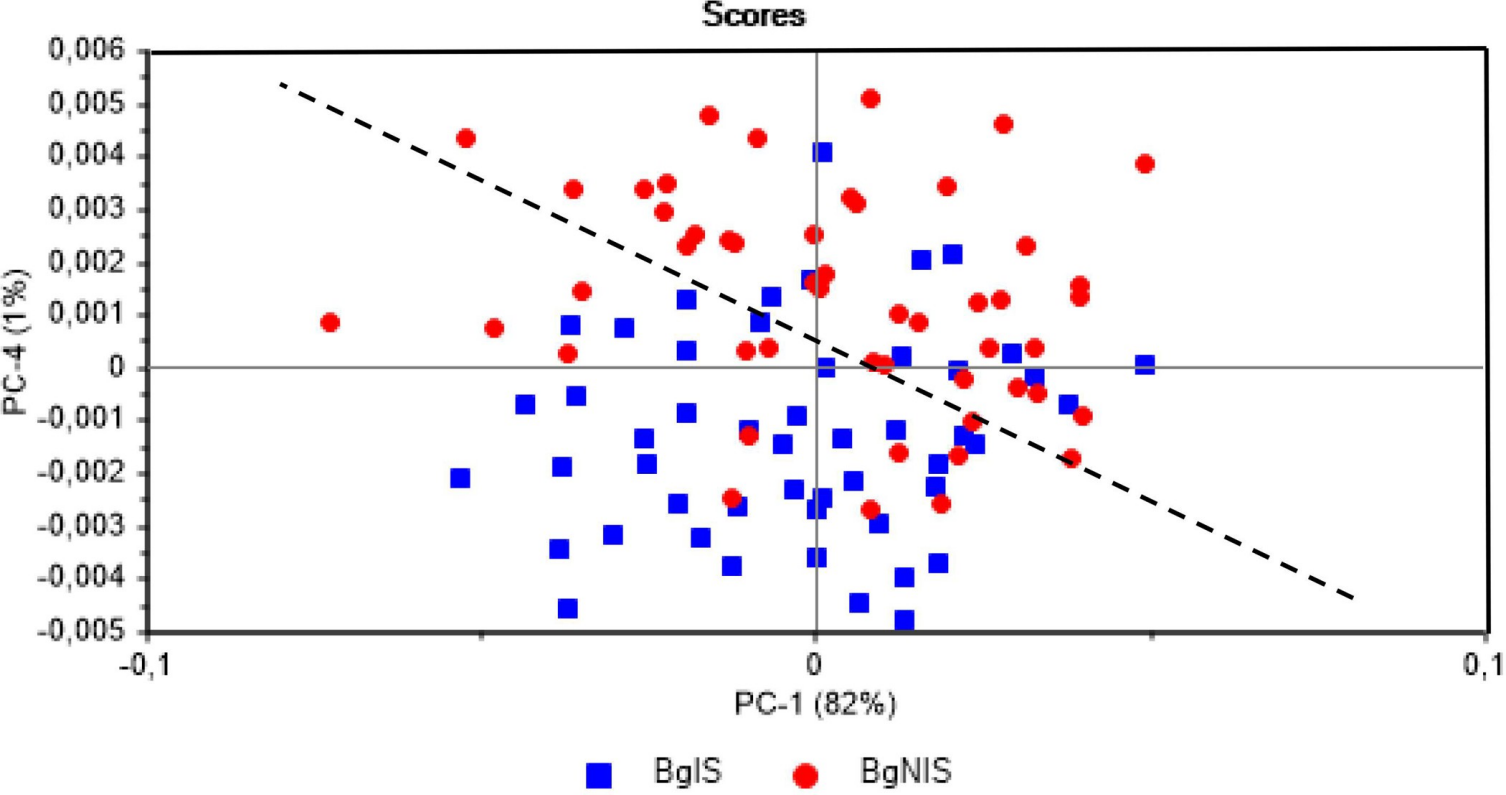
FT-NIR spectra of the parasitological diagnosis. Plot of the scores obtained by Principal Component analysis (PCA) of live *Biomphalaria* snails infected and unexposed to *Schistosoma mansoni*. BgIS = infected *Biomphalaria glabrata*; BgNIS = *Biomphalaria glabrata* not exposed to infection.

The use of FT-NIR as a tool for parasitological diagnosis in alive specimens of *B*. *glabrata* infected by *S*. *mansoni*, allowed the classification and differentiation of infected mollusks from those not exposed to infection. In the PCA evaluation of the data set, it is noted that the scores of PC-4 referring to most infected mollusks assume lower values, while those for mollusks not exposed to *S*. *mansoni* infection show higher values, characterizing the feasibility of classification of the animals into these two groups (Fig 4). PC-4 explains only 1% of the total variance of the pretreated spectral data set and is the principal component retaining the spectral information used to distinguish the infected and non-infected animals. This fact would be expected because most of the spectral variance of the data set is caused by more significant sources such as, the position of the animal in the sampling cell, animal movement inside the cell, among others.

Using LDA a classification method, the overall correct hit rate for the external validation set of samples was 82.5%, with 30 animals from each group (control and infected) randomly selected to compose the modeling set of samples and the 20 others for external validation. A total of seven errors were observed, four of them in relation to infected animals (false negatives). The false negatives could not be associated to the amount of cercariae. Therefore, apparently the number of worms eliminated has no relationship with the classification results obtained using the NIR spectra. Three false positives were obtained from the set of 20 non-infected animals.

These are the first preliminary results obtained using NIR for parasitological diagnosis in *Biomphalaria* spp. Using the same technique, Sikulu-Lord et al. (2016) [6] verified the differentiation of *Aedes aegypti* mosquitoes with endosymbiosis by *Wolbachia* spp. while Fernandes et al. (2018) [8] observed the differentiation of non-infected *A*. *aegypti* from those infected by Zika virus. Maia et al. (2019) [36] performed parasitological diagnosis of *Plasmodium falciparum*-infected mosquitoes of the genus *Anopheles*. The success of differentiating infected mollusks is probably based on the distinct metabolic profile of the animals in face of infection. This, as well as fasting, significantly alters glycogen contents in tissues and its availability in hemolymph [33–35, 37, 38]. Specimens of *B*. *glabrata* infected by *S*. *mansoni* reduces the glycogen contents in the tissues, reaching levels not detectable with biochemical methods, associated with cercariae elimination by the mollusks [33]. Another change is associated with nitrogenous excretion products, due to the change in the urea cycle, provided by the ingestion of arginine by secondary sporocysts to the development of cercariae [34, 35].

In conclusion, the best protocol for the acquisition of NIR spectral data on *Biomphalaria* snails was defined. Shells should be previously (i) dried for a minimum of 15 days at temperature of 26±1°C, and then (ii) placed directly in the equipment probing window with the left side facing the radiation beam. Alive specimens must have, (i) both sides of the shell dried with absorbent paper; (ii) be transferred into a glass vial, and (iii) the vial placed on the apparatus with the left side facing the beam of light.

We highlight that the proposed classification method presented here is innovative aiming at species discrimination using just shells and the possibility of the analysis of alive mollusks. However, the method needs to be further evaluated considering differentiating various species of other mollusk genera. It will require joint efforts from many researchers, who must calibrate and validate the procedures for several different types of living organisms, under various real environmental conditions. The additional data must provide important insights helping the development of a spectrophotometric database of the chemical profile of each living organism and possible chemical changes for different species. We thus emphasize the potential value of NIRS as a useful complementary tool in medical malacology. For the first time, we presented the possibility to differentiate non-infected and infected *B*. *glabrata* by *S*. *mansoni*, using positive snails shedding cercariae. Further studies with infected snails are in progress, using the other intermediate host species.
